# GREM2 inactivation increases trabecular bone mass in mice

**DOI:** 10.1038/s41598-024-63439-4

**Published:** 2024-06-05

**Authors:** Karin H. Nilsson, Petra Henning, Jianyao Wu, Klara Sjögren, Ulf H. Lerner, Claes Ohlsson, Sofia Movérare-Skrtic

**Affiliations:** 1 Department of Internal Medicine and Clinical Nutrition, Institute of Medicine, Sahlgrenska Osteoporosis Centre, Centre for Bone and Arthritis Research at the Sahlgrenska Academy, University of Gothenburg, Gothenburg, Sweden; 2 Department of Drug Treatment, Region Västra Götaland, Sahlgrenska University Hospital, Gothenburg, Sweden

**Keywords:** Bone quality and biomechanics, Endocrine system and metabolic diseases

## Abstract

Osteoporosis is a common skeletal disease affecting millions of individuals world-wide, with an increased risk of fracture, and a decreased quality of life. Despite its well-known consequences, the etiology of osteoporosis and optimal treatment methods are not fully understood. Human genetic studies have identified genetic variants within the *FMN2/GREM2* locus to be associated with trabecular volumetric bone mineral density (vBMD) and vertebral and forearm fractures, but not with cortical bone parameters. GREM2 is a bone morphogenetic protein (BMP) antagonist. In this study, we employed *Grem2*-deficient mice to investigate whether *GREM2* serves as the plausible causal gene for the fracture signal at the *FMN2/GREM2* locus. We observed that *Grem2* is moderately expressed in bone tissue and particularly in osteoblasts. Complete *Grem2* gene deletion impacted mouse survival and body growth. Partial *Grem2* inactivation in *Grem2*^+*/−*^ female mice led to increased trabecular BMD of femur and increased trabecular bone mass in tibia due to increased trabecular thickness, with an unchanged cortical thickness, as compared with wildtype littermates. Furthermore, *Grem2* inactivation stimulated osteoblast differentiation, as evidenced by higher alkaline phosphatase (*Alp*), osteocalcin (*Bglap*), and osterix (*Sp7*) mRNA expression after BMP-2 stimulation in calvarial osteoblasts and osteoblasts from the long bones of *Grem2*^*−/−*^ mice compared to wildtype littermates. These findings suggest that GREM2 is a possible target for novel osteoporotic treatments, to increase trabecular bone mass and prevent osteoporotic fractures.

## Introduction

Osteoporosis is a disease that affects millions of individuals across the globe. One in three men and every other woman will at some point suffer from an osteoporotic fracture, with subsequent great personal suffering and a high societal cost^1^. As the human population is getting increasingly older^2,3^, the number of osteoporosis patients will increase dramatically. While the consequences of this disease are relatively well-known, there remains a knowledge gap concerning its etiology and the most safe and effective treatment methods. In recent years, the development of genome-wide association studies (GWAS) has emerged as a crucial tool for identifying potential therapeutic avenues for osteoporosis^4^.

Separate GWAS meta-analyses for cortical and trabecular volumetric bone mineral density (vBMD) in the tibia have revealed a genetic variant (rs9287237) within the *FMN2/GREM2* locus associated with trabecular vBMD, but not cortical bone parameters^5^. Another GWAS identified a correlated SNP (rs9661787) in the *GREM2* locus as significantly associated with trabecular vBMD in the lumbar spine^6^. The genetic variant (rs9287237) in the *FMN2/GREM2* locus was also associated with fracture risk and X-ray verified vertebral fractures^5^. Furthermore, expression quantitative trait locus (eQTL) analyses in human osteoblasts showed a significant association between the trabecular vBMD associated SNP in the *FMN2/GREM2* locus and the nearby *GREM2* gene's expression, suggesting GREM2 as a potential mediator of the trabecular vBMD association^5^. Notably, most recently, we also demonstrated that the rs9287237 SNP in the *FMN2/GREM2* locus is associated with forearm fractures at a genome-wide significant (GWS) level^7^.

GREM2, also known as Gremlin2 and PRDC (protein related to DAN and cerberus), is a bone morphogenetic protein (BMP) antagonist^8,9^. BMPs have effects in many different tissues, regulating cell proliferation, differentiation, and death^10^, and it has been suggested that BMP signaling is essential for limb bud outgrowth^11^. BMPs regulate proliferation and differentiation of osteoblasts and chondrocytes^8,10^. In vitro studies have demonstrated that reduced *Grem2* expression in osteoblasts elevates osteogenesis and during osteoblast differentiation, BMP-2 induces the expression of *Grem2* that then acts as a negative regulator of BMP-2 signaling^12^.

Despite the compelling evidence from different GWAS, the causal gene responsible for the associations with areal bone mineral density (aBMD), estimated bone mineral density (eBMD, obtained from quantitative ultrasound of the heel), vertebral fractures, and forearm fractures in the *FMN2/GREM2* locus remains to be identified. The objective of this study is to, by using *Grem2*-deficient mice, investigate whether *GREM2* serves as the plausible causal gene for the fracture signal at the *FMN2/GREM2* locus. Our hypothesis posits that *Grem2*-deficient mice will exhibit increased bone mass preferably at trabecular bone sites.

## Materials and methods

### Animal experiment

All animal procedures were approved by the Gothenburg Animal Research Ethics Committee, Sweden, and the animals were cared for according to their and the ARRIVE guidelines. The mice were housed under a controlled temperature (20 °C), and photoperiod (12 h of light and 12 h of darkness), at a humidity of 45–70% in an animal house at University of Gothenburg. The mice were housed in individually ventilated cages (airflow 5 l/h under a balanced pressure) in groups of five or ten. Pellet diet (Envigo Harlan Teklad 2016) and water were available ad libitum.The mice were euthanized using Ketador (Richter Pharma) mixed with Dexdomitor (Orion Pharma), followed by exsanguination and cervical dislocation. Tibia, femur, and vertebra were dissected, fixed in formalin, and stored in ethanol. Soft tissues were snap-frozen in liquid nitrogen following dissection. Cortical bone from cleaned and flushed tibia shafts and trabecular bone from the vertebral body were stored in RNAlater (76106, Qiagen) and frozen.

### Generation of *Grem2-*deficient mice

*Grem2*-deficient mice were generated by breeding female and male *Grem2*^+*/−*^ mice commercially available from Taconic (TF3196, Hudson, NY), on a C57/BL6N background. *Grem2*^+*/*+^ mice were used as littermate controls. In brief, the *Grem2*^−/−^ mice have a deletion of the coding exon 2^13^. Presence of the intact *Grem2* allele was detected using real-time qPCR (StepOnePlus Real-Time PCR System, Thermo Fisher Scientific) with the following primers and FAM-labelled probe: 5′-ATCCTCAACCGCTTCTGCTA-3′, 5′-GGAGTCCTCCTCCTTCTTCA-3′, and 5′-FAM-TCCTTCTACATCCCGCGACACG-3′, whereas absence of the intact *Grem2* allele was detected by the following primers and VIC-labelled probe: 5′-TCTGGATTCATCGACTGTGG-3′, 5′-TCAGCAATATCACGGGTAGC-3′, and 5′-VIC-CCTGATAGCGGTCCGCCACA-3′.

### Assessment of bone parameters

#### Dual X-ray absorptiometry

Total body bone mineral density (BMD) was measured using the Lunar PIXImus densitometer (Wipro GE Healthcare).

#### High-resolution microcomputed tomography (µCT)

High-resolution microcomputed tomography (µCT) analyses were performed on proximal tibia and vertebra L5 using the 1172 µCT (Bruker MicroCT), as previously described^14^. The trabecular bone parameters were analyzed in the metaphyseal region of tibia, starting approximately 650 µm from the proximal growth plate, continuing for roughly 134 µm in distal direction. Cortical thickness was measured in the diaphyseal region of the tibia, starting approximately 5.2 mm from the growth plate, continuing for roughly 134 µm in distal direction. For trabecular parameters from vertebra L5, trabecular bone caudal of the pedicles were analyzed starting at a distance of approximately 4.5 µm caudal of the lower end of the pedicles, continuing in a longitudinal direction for 230 µm in the caudal direction. The data was analyzed using the CTAn software (Bruker MicroCT).

#### Peripheral quantitative computed tomography (pQCT)

Computed tomography (pQCT) analyses were performed using the XCT Research M (v.4.5B, Norland Stratec) at a resolution of 70 µm^15^. In order to measure trabecular volumetric BMD, the scans were positioned in the metaphysis of femur at a distance corresponding to 3.4% of the total femur length, proximal from the distal growth plate. The trabecular region was defined as the inner 45% of the total cross-sectional area^16^.

#### Bone histomorphometry

For dynamic histomorphometry, the mice were labeled intraperitoneally using the fluorochromes calcein (C0875, Merck GmbH) and alizarin (A3882, Merck GmbH) 8 and 1 day prior to termination, respectively. After dissection, tibia was fixated in formalin, dehydrated in ethanol, and imbedded in methyl methacrylate. Dynamic trabecular bone parameters were measured in unstained 8-µm-thick sections, and static trabecular parameters were determined in 4-µm-thick plastic sections stained in Masson-Goldner’s Trichrome. All parameters were analyzed using the OsteoMeasure7 histomorphometry system (OsteoMetrics), according to the guidelines of the American Society for Bone and Mineral Research^17^.

### Real-time quantitative PCR

Total RNA from cortical (shafts from flushed tibia) and trabecular-rich (vertebral body) bone, brain cortex, muscle gastrocnemius, brown fat, retroperitoneal fat, and gonadal fat was prepared using TRIzol Reagent (15596018, Thermo Fisher Scientific) followed by the RNeasy Mini Kit (74116, Qiagen). Total RNA from ovary, hypothalamus, lung, liver, aorta, kidney, spleen, uterus, and heart was prepared using the RNeasy Mini Kit (74116, Qiagen). The RNA was reversed transcribed into cDNA (4368814, Applied Biosystems) and real-time PCR analyses were performed using the StepOnePlus Real-Time PCR System (Thermo Fisher Scientific). *Grem2* expression was measured using the following custom-made primer and probe sets located within the deleted region of exon 2: forward primer: 5′-TGGCTGTGCTGGTAAAGGTA-3′, reverse primer: 5′-TTGATCTGGTGATGCCACCT-3′, and probe: FAM-5′-CGCCCGCAGGCCGGTTCTTC-3′. The following Assay-on-Demand primer and probe sets were used: *Alp*, Mm00475834_m1; *Bglap* (encoding osteocalcin), Mm03413826_mH; and *Sp7* (encoding osterix), Mm04209856_m1. For *Grem2* expression analyses in liver and gonadal fat of *Grem2*^+/−^ heterozygote and wildtype littermates, Mm00501909_m1 Assay-on-Demand primer and probe set was used. Relative gene expression was calculated by the 2^–∆∆Ct^ method using the expression of the 18S ribosomal subunit (4310893E, Thermo Fisher Scientific) as internal standard.

### Primary bone cell cultures

#### Primary calvarial bone cells

Primary calvarial bone cells were isolated from 4 to 6 day old mice by consequential collagenase treatments^18^. Cells in collagenase fractions 6–10 were cultured in complete αMEM (α-MEM medium, 22561-021, Gibco, supplemented with 10% heat inactivated fetal bovine serum (FBS, F7524, Sigma), 2 mM GlutaMAX (35050-038, Gibco), 50 µg/ml gentamicin (15750-037, Gibco), 100 U/ml penicillin and 100 µg/ml streptomycin (15140-148, Gibco)) for 4 days prior to re-seeding at 20,000 cells per square centimeter in 48-well plates. The cells were cultured in complete osteogenic αMEM (complete α-MEM medium with 2 mM β-glycerophosphate (G9422, Sigma), and 0.2 mM l-Ascorbic acid 2-phosphate sesquimagnesium salt hydrate (A8960, Sigma)) with change of half of the media volume every 2–3 days. For mRNA expression analyses, the cells were lysed in RNeasy lysis (RLT) buffer with β-mercaptoethanol (M6250, Sigma-Aldrich) followed with RNeasy Micro RNA kit (74004, Qiagen), cDNA synthesis, and real-time PCR as described above. To study osteoblast differentiation, cells were treated with or without BMP-2 (100 ng/ml, 355-BEC, R&D Systems).

#### Primary osteoblast cell cultures from long bones

Primary osteoblast cell cultures from tibia from 12-week-old female *Grem2*^+*/*+^ and *Grem2*^*−/−*^ mice were isolated by outgrowth of cells from collagenase-treated diaphyseal cortical bone, as previously described^19^. Primary bone cells from tibia were incubated in complete osteogenic αMEM, and the experiments were conducted as described for primary calvarial bone cells above. To study osteoblast differentiation, cells were treated with or without BMP-2 (100 ng/ml, 355-BEC, R&D Systems). For mineralization analyses, the cells were cultured for 10 days and then washed in PBS, fixed in 2.5% glutaric aldehyde in 70% ethanol, washed three times in 70% ethanol and air dried. Mineralized nodules were visualized by staining with 1% alizarin red in 50% ethanol for 5 min, followed by three washes in 50% ethanol and air dried. Whole well stitched images were taken using a Jenoptik Gryphax camera on a Nikon Eclipse 80i microscope. The percentage of the well surface covered by mineralized matrix was determined using the Bioquant Osteo software (version 2023 v23.5.60).

#### Primary osteoclast cell cultures

Bone marrow cells from wildtype mice were cultured in suspension culture discs (430591, Corning) in complete αMEM with 30 ng/ml macrophage-stimulating factor (M-CSF, 416-ML-050, R&D Systems), for 2 days. The adherent bone marrow macrophages (BMMs) were used as osteoclast progenitors^20,21^. The cells were detached and spot-seeded in 24-well plates (20,000 cells per well). The cells were cultured in complete αMEM with 30 ng/ml M-CSF with or without the addition of 4 ng/ml receptor activator of nuclear factor κ-B ligand (RANKL, 462-TEC, R&D Systems) to induce osteoclast differentiation. After 3 days, the medium was changed, and 4 days after seeding, the cells were harvested for RNA preparation by lysis in RLT buffer with β-mercaptoethanol.

### Statistics

Results are presented as mean ± standard error of the mean. Genotype ratios were tested against a Mendelian ratio (1:2:1) with a one sample chi-square goodness of fit test (SPSS, version 29.0.0.0). One-way ANOVA followed by Dunnett’s multiple comparisons test (GraphPad Prism, version 10.0.3) was used to assess the effect of genotype on body weight between *Grem2*^*−/−*^ versus *Grem2*^+*/*+^ and *Grem2*^+*/−*^ versus *Grem2*^+*/*+^ mice. To calculate the difference in *Grem2* expression over time in wildtype calvarial osteoblast, one-way ANOVA followed by Dunnett’s multiple comparisons test (GraphPad Prism) was used, using the expression of day 1 as the reference. To evaluate the effect of genotype (*Grem2*^+*/−*^ and *Grem2*^+*/*+^), age (10 or 12 weeks), and the interaction thereof, two-way ANOVA was used (GraphPad Prism). For analyses of primary osteoblast cell cultures, Student’s *t* test was used to evaluate the difference in gene expression between two groups (*Grem2*^+/+^ mice vs. *Grem2*^+/−^ mice, *Grem2*^+*/*+^ cells vs. *Grem2*^*−/−*^ cells, or Ctrl vs. BMP-2). Two-way ANOVA (GraphPad Prism) was used to assess overall interaction between BMP-2 effect in *Grem2*^+*/*+^ cells vs. BMP-2 effect in *Grem2*^*−/−*^ cells. Power analysis was performed to determine group sizes. The analysis suggested that the use of eleven *Grem2*^+*/*+^ and eleven *Grem2*^+*/−*^ mice would give 80% power to detect a biological significant effect (where *P* < 0.05) with a 1.26 s.d. change in trabecular bone mass. We therefore aimed to use at least eleven mice per group in the in vivo studies.

## Result

### *Grem2* is moderately expressed in bone tissue

**Figure 1:**
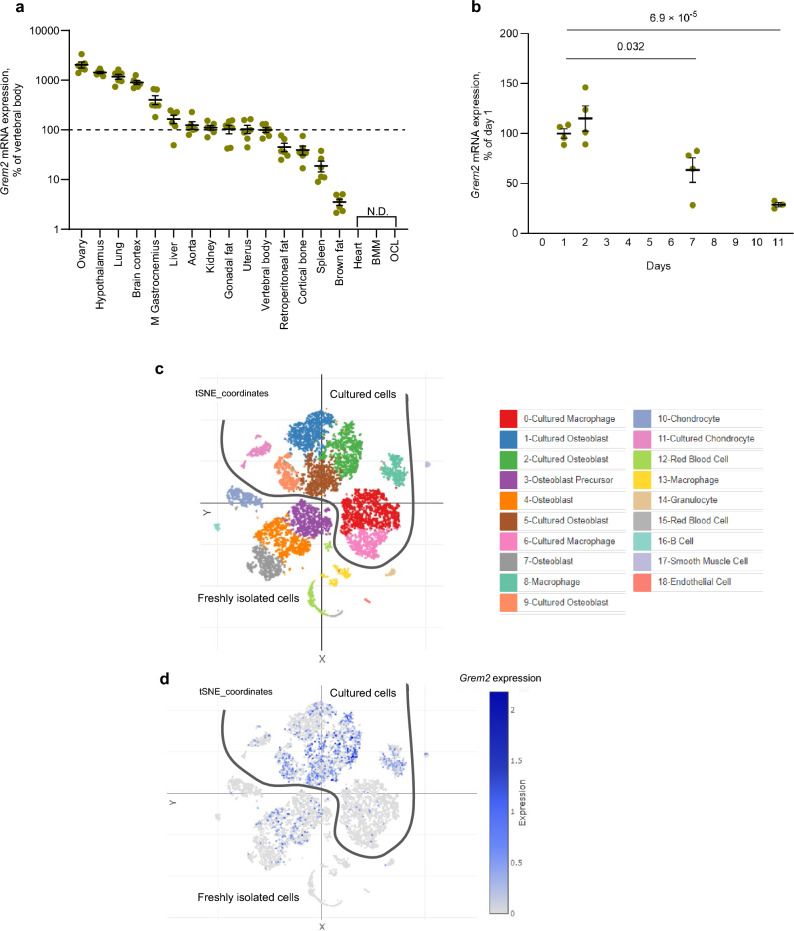
*Grem2* is expressed in osteoblasts but not in osteoclasts in mice. (**a**) *Grem2* mRNA expression pattern in various tissues of 12-week-old female, wildtype mice (n = 6), bone marrow macrophages (BMM), and osteoclasts (OCL). Data presented as % of the expression in trabecular-rich vertebral body and shown as individual values with the mean presented as horizontal lines and ± standard error of the mean as vertical lines. *N.D.* not detectable. (**b**) *Grem2* mRNA expression in primary calvarial osteoblasts from wildtype mice decreases over time in culture. Data presented as % of the expression at day 1 and shown as individual values with the mean presented as horizontal lines and ± standard error of the mean as vertical lines. Difference in expression over time was analyzed using one-way ANOVA followed by Dunnett’s multiple comparisons test with day 1 as the reference group. n = 4 per timepoint. The experiment was repeated three times. (**c,d**) Cluster analysis (**c**), and feature plots of *Grem2* expression (**d**) in freshly isolated primary mouse calvarial cells and cultured calvarial cells published by Ayturk et al.^22^, publicly available at Single Cell Portal (https://singlecell.broadinstitute.org/single_cell/study/SCP1337). Freshly isolated cells and cultured cells form separate clusters as indicated in the figure^22^.

To study the expression pattern of *Grem2*, we compared the *Grem2* expression in 16 tissues from mice (female, aged 12 weeks) and observed high expression in ovary, certain brain structures, such as hypothalamus and brain cortex, and lung, and moderate expression in cortical bone and vertebral body, rich in trabecular bone (Fig. 1a). Analyses of isolated calvarial cells, containing a large proportion of osteoblasts, indicated that cultured primary osteoblasts express *Grem2,* but the expression decreases with time in these cultures (Fig. 1b). No expression of *Grem2* was present in cultured bone marrow macrophages (BMM) or RANKL-differentiated osteoclasts (OCL) (Fig. 1a). To further elucidate expression of *Grem2* in bone cells, single cell RNA-sequencing (scRNAseq) datasets of freshly isolated primary mouse calvarial cells and cultured calvarial cells were used^22^. Analysis of the datasets demonstrated expression of *Grem2* both in freshly isolated primary osteoblasts and in cultured osteoblasts from the calvaria (Fig. 1c, d). *Grem2* was modestly expressed in osteoblasts in scRNAseq datasets of mouse bone marrow cells (Supplementary Fig. 1)^23^. *FMN2* is expresssed in brain tissues but no expression of *FMN2* was observed in freshly isolated primary mouse calvarial cells or in cultured calvarial cells (Supplementary Fig. 2).

### Complete *Grem2* gene deletion affects mouse survival and growth

To obtain *Grem2*-deficient mice*,* we bred *Grem2*^+*/−*^ female mice with *Grem2*^+*/−*^ male mice. We observed that *Grem2*^*−/−*^ mice were not born according to Mendel’s law of inheritance with the expected Mendelian ratio (1:2:1). From 320 pups, we obtained 88 *Grem2*^+*/*+^ mice, 200 *Grem2*^+*/−*^ mice, and 32 *Grem2*^*−/−*^ mice, demonstrating that a complete deletion of the *Grem2* gene affects survival (Supplementary Fig. 3). A one sample chi-square goodness of fit test confirmed an unexpected Mendelian ratio (*P* = 2.5 × 10^–9^).

At 5 weeks of age, the few *Grem2*^*−/−*^ mice born were small with a lower body weight compared to wildtype littermates (female mice: -13.3 ± 5.3%, *P* = 0.0046; male mice: −20.6 ± 5.8%, *P* = 3.0 × 10^–4^). No differences in body weight were found between *Grem2*^+*/−*^ heterozygote and wildtype littermates (female mice: −2.6 ± 1.5%, *P* = 0.74; male mice: + 1.6 ± 1.9%, *P* = 0.88).

### Increased trabecular bone mass in female mice with partial *Grem2* inactivation

**Figure 2:**
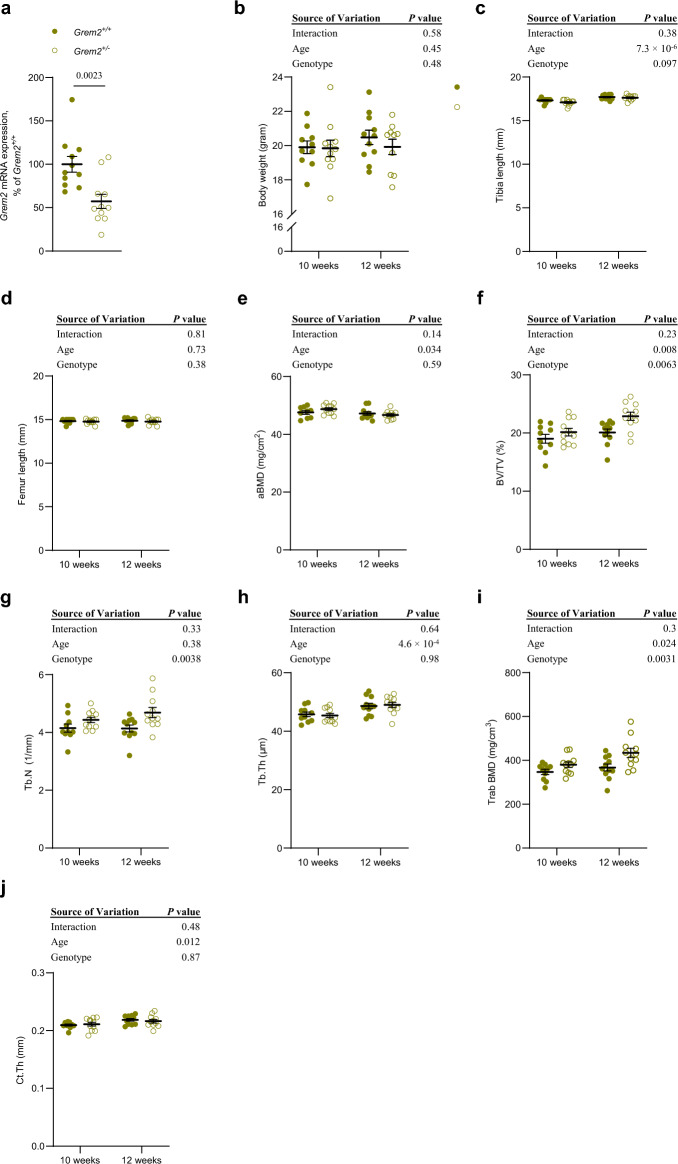
Increased trabecular bone mass in mice with partial inactivation of *Grem2.* (**a**) mRNA expression analyses of *Grem2* in trabecular-rich vertebral body of 12-week-old male *Grem2*^+*/*+^ (n = 11) and *Grem2*^+*/−*^ (n = 11) mice. (**b**) Body weight in *Grem2*^+*/*+^ (10-week-old, n = 10; 12-week-old, n = 11), and *Grem2*^+*/−*^ (10-week-old, n = 11; 12-week-old, n = 10) mice. (**c,d**) Length of tibia (**c**) and femur (**d**) in *Grem2*^+*/*+^ (10-week-old, n = 10; 12-week-old, n = 11), and *Grem2*^+*/−*^ (10-week-old, n = 11; 12-week-old, n = 11) mice. (**e**) Whole body areal bone mineral density (aBMD) as measured by DXA in *Grem2*^+*/*+^ (10-week-old, n = 9; 12-week-old, n = 11), and *Grem2*^+*/−*^ (10-week-old, n = 11; 12-week-old, n = 11) mice. (**f–h**) Trabecular bone volume over tissue volume (BV/TV (**f**)), trabecular number (Tb.N (**g**)), and trabecular thickness (Tb.Th (**h**)) in tibia as measured by µCT in *Grem2*^+*/*+^ (10-week-old, n = 10; 12-week-old, n = 11), and *Grem2*^+*/−*^ (10-week-old, n = 11; 12-week-old, n = 11) mice. (**i**) Trabecular bone mineral density (BMD) in femur as measured by pQCT in *Grem2*^+*/*+^ (10-week-old, n = 10; 12-week-old, n = 11), and *Grem2*^+*/−*^ (10-week-old, n = 11; 12-week-old, n = 11) mice. (**j**) Cortical thickness of tibia as measured by µCT in *Grem2*^+*/*+^ (10-week-old, n = 10; 12-week-old, n = 11), and *Grem2*^+*/−*^ (10-week-old, n = 11; 12-week-old, n = 11) mice. Unless otherwise stated, the results refer to female mice. Individual values are presented in all graphs with the mean presented as horizontal lines and ± standard error of the mean presented as vertical lines. Statistical analyses were performed using two-sided Student’s *t* test (for (**a**)), and a mixed model two-way ANOVA were used to evaluate the genotype effect (*Grem2*^+*/*+^ or *Grem2*^+*/−*^), the age effect (10-week-old or 12-week-old), and the overall interaction effect between genotype and age (for (**b–j**)).

As the *Grem2*^*−/−*^ mice born were severely growth restricted, it was not feasible to study their skeletal phenotype. Therefore, skeletal analyses on adult mice were performed only on the *Grem2*^+*/−*^ heterozygote and wildtype littermates. *Grem2* mRNA expression analysis demonstrated a reduction in *Grem2* expression in the trabecular-rich vertebral body (−42.8 ± 8.2%, *P* = 0.0023) in the heterozygote *Grem2*^+*/−*^ mice, compared with wildtype *Grem2*^+*/*+^ littermates (Fig. 2a). A similar decrease in *Grem2* expression was seen in liver and gonadal fat (Supplementary Fig. 4).

Similar to their condition at 5 weeks of age, 10- and 12-week-old *Grem2*^+*/−*^ female mice appeared healthy when compared to *Grem2*^+*/*+^ littermate controls, with unchanged body weight (−1.5 ± 2.15%, *P* = 0.48; Fig. 2b). Furthermore, when analyzing the skeleton, *Grem2*^+*/−*^ female mice had a similar length of long bones compared to *Grem2*^+*/*+^ littermate controls (tibia; −8.86 ± 0.51%, *P* = 0.10, Fig. 2c; femur; −0.54 ± 0.60%, *P* = 0.38, Fig. 2d). DXA analyses of the two independent 10- and 12-week-old *Grem2*^+*/−*^ female mice cohorts compared to *Grem2*^+*/*+^ littermate controls revealed no differences in total body areal bone mineral density (aBMD; + 0.63 ± 1.14%, *P* = 0.59; Fig. 2e). However, a more detailed analysis of the tibia by μCT revealed an increase in trabecular bone volume fraction (BV/TV, + 10.0 ± 3.5%, *P* = 0.0063; Fig. 2f), as a result of increased trabecular number (Tb.N, + 10.6 ± 3.3%, *P* = 0.0038; Fig. 2g), whereas the trabecular thickness was unchanged (Tb.Th, −0.04 ± 1.80%, *P* = 0.98; Fig. 2h). pQCT analysis of femur displayed an increase in trabecular BMD in the *Grem2*^+*/−*^ female mice cohorts compared to the *Grem2*^+*/*+^ littermate controls (+ 14.0 ± 4.5%, *P* = 0.0031; Fig. 2i), confirming the results from tibia. In contrast, there was no difference in the trabecular bone of the vertebra in the *Grem2*^+*/−*^ female mice compared to *Grem2*^+*/*+^ littermate controls (Table 1). No difference in cortical thickness of the long bones was observed in the *Grem2*^+*/−*^ female mice compared to *Grem2*^+*/*+^ littermate controls (Ct.Th, −0.19 ± 1.26%, *P* = 0.87; Fig. 2j). In order to determine the mechanisms for the increased trabecular BV/TV of the tibia, static and dynamic histomorphometry were performed. However, static histomorphometry did not reveal any effects on neither the number of osteoblasts nor the number of osteoclasts and the dynamic histomorphometry analyses of the trabecular bone of tibia did not reveal any differences in the bone formation rate in the *Grem2*^+/−^ mice compared to *Grem2*^+/+^ littermate controls (Table 2), suggesting a new steady state of bone turnover has been reached.

**Table 1:** Trabecular parameters in vertebra L5.

	10 weeks		12 weeks		Two-way ANOVA		
	*Grem2*^+*/*+^	*Grem2*^+*/−*^	*Grem2*^+*/*+^	*Grem2*^+*/−*^	*P* interaction	*P* age	*P* genotype
Bone volume over tissue volume (BV/TV, %)	20.7 ± 0.62	22.6 ± 0.64	23.1 ± 0.76	22.5 ± 0.66	0.07	0.10	0.33
Trabecular number (Tb.N, 1/mm)	4.70 ± 0.19	5.14 ± 0.13	5.67 ± 0.14	5.53 ± 0.18	0.08	1.2 × 10^–4^	0.35
Trabecular thickness (Tb.Th, µm)	44.2 ± 0.80	44.0 ± 0.63	40.7 ± 1.14	40.7 ± 0.69	0.91	2.8 × 10^–4^	0.89

µCT measurements of trabecular parameters in vertebra L5 of female mice. Values are given as mean ± standard error of the mean in *Grem2*^+*/*+^ (10-week-old, n = 10; 12-week-old, n = 11), and *Grem2*^+*/−*^ (10-week-old, n = 11; 12-week-old, n = 11) mice. A mixed model two-way ANOVA was used to evaluate the genotype effect (*Grem2*^+*/*+^ or *Grem2*^+*/−*^), the age effect (10-week-old or 12-week-old), and the overall interaction effect between genotype and age.

**Table 2:** Histomorphometry analyses of tibia in 12-week-old female mice.

	*Grem2*^+*/*+^	*Grem2*^+*/−*^	*P* value
*Static histomorphometry*			
Number of osteoclasts per trabecular bone perimeter (N.Oc/B.Pm; mm^−1^)	1.46 ± 0.21	1.76 ± 0.22	0.33
Osteoclast surface per trabecular bone surface (Oc.S/BS; %)	3.68 ± 0.53	4.67 ± 0.53	0.21
Number of osteoblasts per trabecular bone perimeter (N.Ob/B.Pm; mm^−1^)	10.7 ± 0.92	10.2 ± 0.86	0.71
Osteoblast surface per trabecular bone surface (Ob.S/BS; %)	17.7 ± 1.84	17.7 ± 1.73	0.99
*Dynamic histomorphometry*			
Mineralizing surface per trabecular bone surface (MS/BS; %)	47.5 ± 1.84	47.9 ± 1.49	0.91
Mineral apposition rate (MAR; µm/day)	1.74 ± 0.07	1.75 ± 0.09	0.96
Bone formation rate per tissue volume (BFR/TV; %/year)	171 ± 16.6	183 ± 14.3	0.59
Bone formation rate per trabecular bone surface (BFR/BS; µm^3^/µm^2^/year)	299 ± 20.9	304 ± 14.4	0.87

Bone histomorphometry analyses of trabecular bone in tibia in 12-week-old female *Grem2*^+*/*+^ (n = 10) and *Grem2*^+*/−*^ (n = 11) mice. A two-tailed Student’s *t* test was used to evaluate the difference between the groups.

Adult 10-week-old *Grem2*^+*/−*^ male mice also had unchanged body weight and length of the long bones as compared to their *Grem2*^+*/*+^ littermate controls (Table 3). DXA analysis revealed an increased aBMD in the *Grem2*^+*/−*^ male mice compared to *Grem2*^+*/*+^ littermate controls (+ 5.36 ± 1.3%, *P* = 0.011; Table 3). When the trabecular and cortical bone was analyzed separately, an increasing trend in BV/TV was noticed (+ 9.42 ± 7.31%, *P* = 0.28), however, no other differences was detected (Table 3).

**Table 3:** Bone parameters of 10-week-old male mice.

	*Grem2*^+*/*+^	*Grem2*^+*/−*^	*P* value
Body weight (g)	25.8 ± 0.4	26.7 ± 0.8	0.32
Tibia length (mm)	18.0 ± 0.1	17.8 ± 0.1	0.27
Femur length (mm)	15.3 ± 0.1	15.4 ± 0.1	0.62
*DXA*			
Areal bone mineral density (mg/cm^3^)	49.0 ± 0.7	51.7 ± 0.6	0.01
*µCT*			
Bone volume over tissue volume (%)	23.8 ± 1.1	26.0 ± 1.7	0.28
Trabecular number (1/mm)	5.15 ± 0.2	5.38 ± 0.3	0.51
Trabecular thickness (µm)	46.2 ± 0.7	48.3 ± 1.5	0.19
Cortical thickness (µm)	235 ± 4.5	238 ± 5.3	0.60

Whole-body DXA analyses and µCT analyses of tibia in male 10-week-old mice. All values are given as mean ± standard error of the mean. *Grem2*^*+/+*^ mice, n = 12; *Grem2*^+*/−*^, n = 10. A two-tailed Student’s *t* test was used to evaluate the difference between *Grem2*^+*/*+^ and *Grem2*^+*/−*^ mice.

### *Grem2* inactivation stimulates osteoblast differentiation in primary bone cells

**Figure 3:**
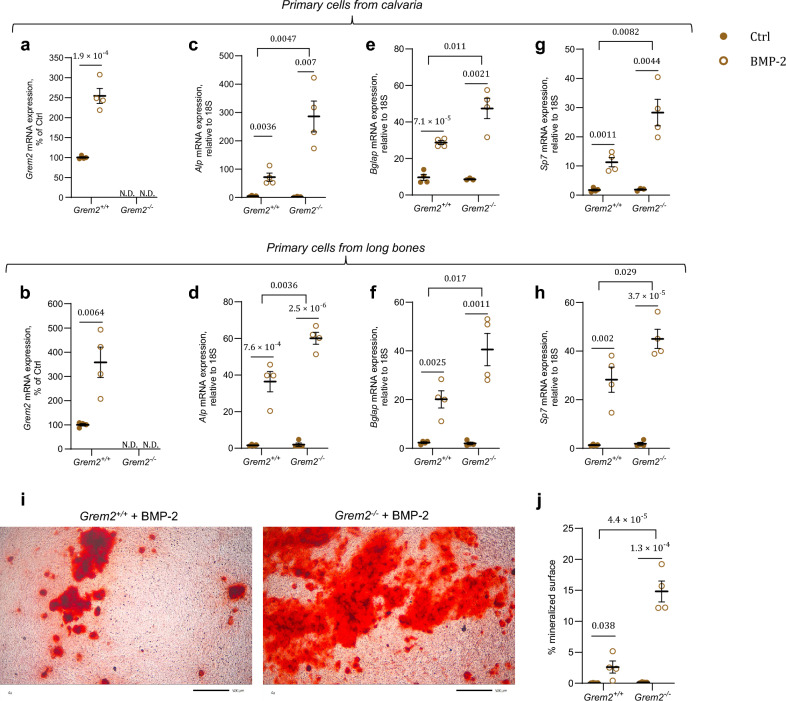
*Grem2* inactivation stimulates osteoblast differentiation in primary bone cells. (**a,b**) mRNA expression levels of *Grem2* in primary calvarial bone cells (**a**) and primary cells from long bones (**b**), after being cultured in osteogenic media and stimulated with or without bone morphogenetic protein-2 (BMP-2) for 7 days. (**c,d**) mRNA expression levels of *Alp* in primary calvarial bone cells (**c**) and primary cells from long bones (**d**), after being cultured in osteogenic media and stimulated with or without BMP-2 for 7 days. (**e,f**) mRNA expression levels of *Bglap* in primary calvarial bone cells (**e**) and primary cells from long bones (**f**), after being cultured in osteogenic media and stimulated with or without BMP-2 for 7 days. (**g,h**) mRNA expression levels of *Sp7* in primary calvarial bone cells (**g**) and primary cells from long bones (**h**), after being cultured in osteogenic media and stimulated with or without BMP-2 for 7 days. (**i**) Alizarin red staining of mineralized nodules in osteoblast cultures of long bones from *Grem2*^+*/*+^ (left) and *Grem2*^*−/−*^ (right) mice, after being cultured in osteogenic media and stimulated with BMP-2 for 10 days. Scale bar: 500 µm. (**j**) Quantification of mineralized nodules stained with alizarin red in osteoblast cultures of long bones from *Grem2*^+*/*+^ and *Grem2*^*−/−*^ mice, after being cultured in osteogenic media and stimulated with or without BMP-2 for 10 days. Individual values are presented in all graphs with the mean presented as horizontal lines and ± standard error of the mean presented as vertical lines. Statistical analyses were performed using two-sided Student’s *t* test when comparing two groups (*Grem2*^+*/*+^ vs. *Grem2*^*−/−*^, or Ctrl vs. BMP-2 treatment), and a mixed model two-way ANOVA was used to evaluate the overall interaction effect between BMP-2 treatment in *Grem2*^+*/*+^ cells vs. BMP-2 treatment in *Grem2*^*−/−*^ cells.

As GREM2 is an inhibitor of BMP, we aimed to investigate if downregulation of *Grem2* affects osteoblast differentiation*.* To this end, we harvested primary calvarial bone cells from *Grem2*^+*/*+^ and *Grem2*^*−/−*^ pups, and primary osteoblast cells from long bones of *Grem2*^+*/*+^ and *Grem2*^*−/−*^ mice. As anticipated, the calvarial osteoblasts and osteoblasts isolated from long bones of *Grem2*^*−/−*^ mice cultured in osteogenic media, did not display any *Grem2* expression (Fig. 3a, b). Calvarial osteoblasts isolated from wildtype mice, cultured in osteogenic media and stimulated with BMP-2 for 7 days, showed an upregulation in *Grem2* expression (+ 154 ± 18.9%, *P* = 1.9 × 10^–4^; Fig. 3a). Similarly, an increase in *Grem2* expression was observed in BMP-2 stimulated osteoblasts isolated from long bones of wildtype mice (+ 258 ± 62.8%, *P* = 0.0064; Fig. 3b). As expected, BMP-2 stimulation had no effect on *Grem2* expression in calvarial osteoblasts or osteoblasts isolated from the long bones of *Grem2*^*−/−*^ mice (Fig. 3a, b).

To evaluate the role of GREM2 in osteoblast differentiation, we examined the expression levels of *Alp*, *Bglap* (which encodes osteocalcin), and *Sp7* (which encodes osterix). Upon stimulation with BMP-2 for 7 days, both calvarial osteoblasts and osteoblasts from the long bones of wildtype mice exhibited a noteworthy increase in *Alp* (+ 1448 ± 311%, *P* = 0.0036, Fig. 3c; and + 2131 ± 339%, *P* = 7.6 × 10^–4^, Fig. 3d, respectively), *Bglap* (+ 197 ± 11.7%, *P* = 7.1 × 10^–5^, Fig. 3e; and + 765 ± 152%, *P* = 0.0025, Fig. 3f, respectively), and *Sp7* expression (+ 537 ± 89.3%, *P* = 0.0011, Fig. 3g; and + 2049 ± 392%, *P* = 0.002, Fig. 3h, respectively). Similarly, calvarial osteoblasts and osteoblasts from the long bones of *Grem2*^*−/−*^ mice also demonstrated a substantial increase in *Alp* (+ 299 ± 75.9%, *P* = 0.007, Fig. 3c; and + 2917 ± 163%, *P* = 2.5 × 10^–6^, Fig. 3d, respectively), *Bglap* (+ 447 ± 64.5%, *P* = 0.0021, Fig. 3e; and + 1910 ± 328, *P* = 0.0011, Fig. 3f, respectively), and *Sp7* (+ 1366 ± 235%, *P* = 0.0044, Fig. 3g; and + 2296 ± 211%, *P* = 3.7 × 10^–5^, Fig. 3h, respectively) expression following the same BMP-2 stimulation period. Notably, the impact of BMP-2 stimulation on *Alp*, *Bglap*, and *Sp7* expression was more pronounced in cells from *Grem2*^*−/−*^ mice compared to those from *Grem2*^+*/*+^ littermates, as evident by interaction effects in both calvarial osteoblasts (*Alp*; F (1,11) = 12.5, *P* = 0.0047, *Bglap*; F (1,11) = 9.34, *P* = 0.011, and *Sp7*; F (1,11) = 10.4, *P* = 0.0082) and osteoblasts from the long bones (*Alp*; F (1,12) = 12.7, *P* = 0.0036, *Bglap*; F (1,12) = 7.59, *P* = 0.017, and *Sp7*; F (1,12) = 6.17, *P* = 0.029). To study mineralization, osteoblasts derived from long bones were stimulated with or without BMP-2 for 10 days and then stained with alizarin red (Fig. 3i, Supplementary Fig. 5). The percentage of mineralized surface increased following BMP-2 stimulation in osteoblasts from both *Grem2*^+*/*+^ (+ 6867 ± 2569%, *P* = 0.038) and *Grem2*^*−/−*^ mice (+ 11,522 ± 1320%, *P* = 1.3 × 10^–4^) (Fig. 3j). However, the stimulatory response on mineralized surface was more pronounced in cells from *Grem2*^*−/−*^ mice compared to those from littermate wildtype mice (interaction effect, F (1,12) = 38.81, *P* = 4.4 × 10^–5^).

## Discussion

The *FMN2/GREM2* locus was previously recognized as a bone-related locus through GWAS^5^. This study aimed to determine whether *GREM2* functions as the plausible causal gene underlying the fracture signal within the *FMN2/GREM2* locus. Utilizing a globally *Grem2*-deficient mouse model in our study, we scrutinized the model's phenotype, revealing evidence that establishes *GREM2* as the plausible causative gene for the fracture signal at the *FMN2/GREM2* locus. Primary bone cell cultures derived from *Grem2*^*−/−*^ mice demonstrated a greater increase in *Alp, Bglap,* and *Sp7* expression following BMP-2 stimulation compared to wildtype cells, indicating an increase in osteoblast differentiation as a result of the GREM2 inactivation.

By using mRNA expression analyses and publicly available databases containing analyses from single cell RNA sequencing data, we demonstrated that *Grem2* is expressed in several tissues and cell types including osteoblasts but not osteoclasts. These results are consistent with a previous report indicating expression of *Grem2* in pre-osteoblasts of embryonic day 18.5 mouse calvaria^12^, and our previous study, demonstrating *GREM2* expression in human osteoblasts^5^. The expression of *Grem2* in calvarial bone cells decreased over time in osteogenic media. However, since the time course gene expression analyses were made in total RNA preparations it cannot be concluded if the expression per osteoblast is changed or if the relative number of osteoblasts in the cultures decrease compared to other cells in the primary cell cultures. Several studies have shown that the percentage of macrophages increase during in vitro culture of calvarial bone cells ^22,24^.

The low number of born *Grem2*^*−/−*^ mice implies that GREM2 directly affects crucial developmental processes. This observation is in line with a previous indication from the International Mouse Phenotyping Consortium (IMPC), suggesting preweaning lethality of *Grem2*^*−/−*^ mice^25^. A previous study in zebrafish^26^ demonstrated that loss of *grem2* leads to overexpression of *pitx2* and downregulation of *lefty2*, two crucial regulators of asymmetric cardiac development^27,28^. Moreover, *grem2* knockdown in zebrafish increases the levels of activated phosphorylated Smad and BMP signaling within cardiac cells, interfering with both ventricular and atrial differentiation, indicating that GREM2 is required for pharyngeal mesoderm patterning, proper cardiac differentiation, and atrial chamber formation during cardiac development^26^. Thus, the low number of *Grem2* knockout mice born may be attributed to the pivotal role of GREM2 in cardiac development. Furthermore, the few *Grem2*^*−/−*^ mice born in the present study were growth restricted, which might also be a consequence of impaired cardiac development. Given the growth restriction in *Grem2*^*−/−*^ mice, further skeletal analyses were focused on *Grem2*^+*/−*^ heterozygote mice and their wildtype littermates.

The *Grem2*^+*/−*^ mice appeared healthy, with no differences in body weight or long bone length compared to wildtype controls. Detailed skeletal analyses using DXA and CT scans revealed intriguing findings. In female mice, no differences were observed in total body areal bone mineral density (aBMD), but *Grem2*^+*/−*^ mice displayed an increase in trabecular BMD of femur and trabecular BV/TV of the tibia, attributed to an elevated trabecular number, whereas there was no change in cortical thickness.

As we previously demonstrated that a signal within the *FMN2/GREM2* locus was associated with trabecular but not cortical bone parameters in humans^5^, our present findings, together with the report on large-scale whole exome sequencing data in the UK Biobank dataset revealing that missense variants in the *GREM2* gene are strongly associated with estimated BMD in the heel^29^, support the notion that it is plausible that the *GREM2* gene within this locus is linked to trabecular bone parameters^5^. Furthermore, our results, demonstrating that GREM2 is important for trabecular but not cortical bone parameters of the long bones, is in accordance with the fact that a signal within the *FMN2/GREM2* locus has been linked to forearm fractures^7^, as it was recently demonstrated that distal forearm fractures are highly dependent on trabecular BMD^30^.

As neither static nor dynamic histomorphometric analyses provided insights into the significance of GREM2 for the number of osteoclasts or osteblasts, or its impact on the bone formation process, possibly due to the establishment of a new equilibrium within the bone tissue in mice having a reduced expression of GREM2 from birth, additional in vitro studies were conducted. GREM2 is a BMP antagonist and we showed that stimulating calvarial osteoblasts and osteoblasts derived from the long bones with BMP-2, resulted in extensively enhanced expression of *Grem2*. These findings confirm and extend previous findings from both pre-osteoblasts from embryonic mouse calvaria and human bone marrow-derived mesenchymal stem cells among others, demonstrating an upregulation of *Grem2* expression by BMP-2 stimulation^12,31–33^. These findings emphasize that expression of *Grem2* is involved in a negative feedback mechanism. Importantly, the increase in expression of the osteogenic markers *Alp*, *Bglap*, and *Sp7* was more pronounced in BMP-2 stimulated osteoblasts, from either calvaria or long bones, derived from *Grem2*-inactivated mice, further emphasizing the functional role of GREM2 as a BMP-2 antagonist. These results are in line with results from previous reports demonstrating that silencing *Grem2* expression in cell cultures increases expression of *Alp* after BMP-2 stimulation, whereas overexpression of *Grem2* in cell cultures decreases expression of *Alp*^12,32^. Mineralization studies on osteoblasts derived from long bones further confirmed the effect of *Grem2* on osteoblast differentiation, as cells from mice lacking *Grem2* had a higher percentage of mineralized surface after stimulation with BMP-2, when compared to cells from wildtype mice. Together with previous studies, our results demonstrate that suppression of *Grem2* promotes osteoblast differentiation. The *Grem2*-deficient mouse model used in this study only captures changes in gene expression and does not account for other potential impacts, such as altered protein conformation or functional changes, which is a limitation of the study. Another limitation of the present study is that no data of *FMN2* knockout mice are included. However, single cell RNA sequencing data demonstrated no expression of *FMN2* in either freshly isolated primary mouse calvarial cells or cultured calvarial cells. Furthermore, a recent report on large-scale whole exome sequencing data in the UK Biobank dataset revealed that missense variants in the *GREM2* gene, but not in the *FMN2* gene, are strongly associated with estimated BMD in the heel, supporting the notion that GREM2 and not FMN2 is crucial for bone mass regulation^29^.

In conclusion, we functionally show that a partial deletion of *Grem2* in mice leads to an increase in trabecular bone mass of long bones, with unchanged cortical bone. By using two different primary osteoblast cell cultures, we report mechanistic data showing that *Grem2*-inactivated cells display enhanced response to BMP-2 stimulation on osteoblast differentiation, as evident by an augmented increase in *Alp, Bglap*, and *Sp7* expression. We believe that GREM2, being a BMP antagonist, is a promising new target for osteoporosis treatment. Further studies are warranted to explore the impact of GREM2 on bone mass and strength in neonatal and aged mice, and utilizing cell-specific and inducible knockout mouse models will provide a more comprehensive understanding of GREM2's involvement in bone biology.

### Supplementary Information

Supplementary Figures.

## Data Availability

The datasets generated and analysed in the present study are available from the corresponding author upon reasonable request.
