# Supplementary material for: GREM2 inactivation increases trabecular bone mass in mice
Source: Sci Rep. 2024 Jun 5;14:12967. doi: 10.1038/s41598-024-63439-4 (PMC11153596; doi:10.1038/s41598-024-63439-4)
Supplement: Supplementary file 1 — Supplementary Figures. [file 41598_2024_63439_MOESM1_ESM.pdf]

Supplementary Figures

GREM2 inactivation increases trabecular bone mass in mice

Karin H. Nilsson[1*], Petra Henning[1], Jianyao Wu[1], Klara Sjögren[1], Ulf H. Lerner[1], Claes Ohlsson[1,2], Sofia Movérare-Skrtic[1]

[1]Department of Internal Medicine and Clinical Nutrition, Institute of Medicine, Sahlgrenska Osteoporosis Centre, Centre for Bone and Arthritis Research at the Sahlgrenska Academy, University of Gothenburg, Gothenburg, Sweden

[2]Region Västra Götaland, Sahlgrenska University Hospital, Department of Drug Treatment, Gothenburg, Sweden

Supplementary Figure 1

a

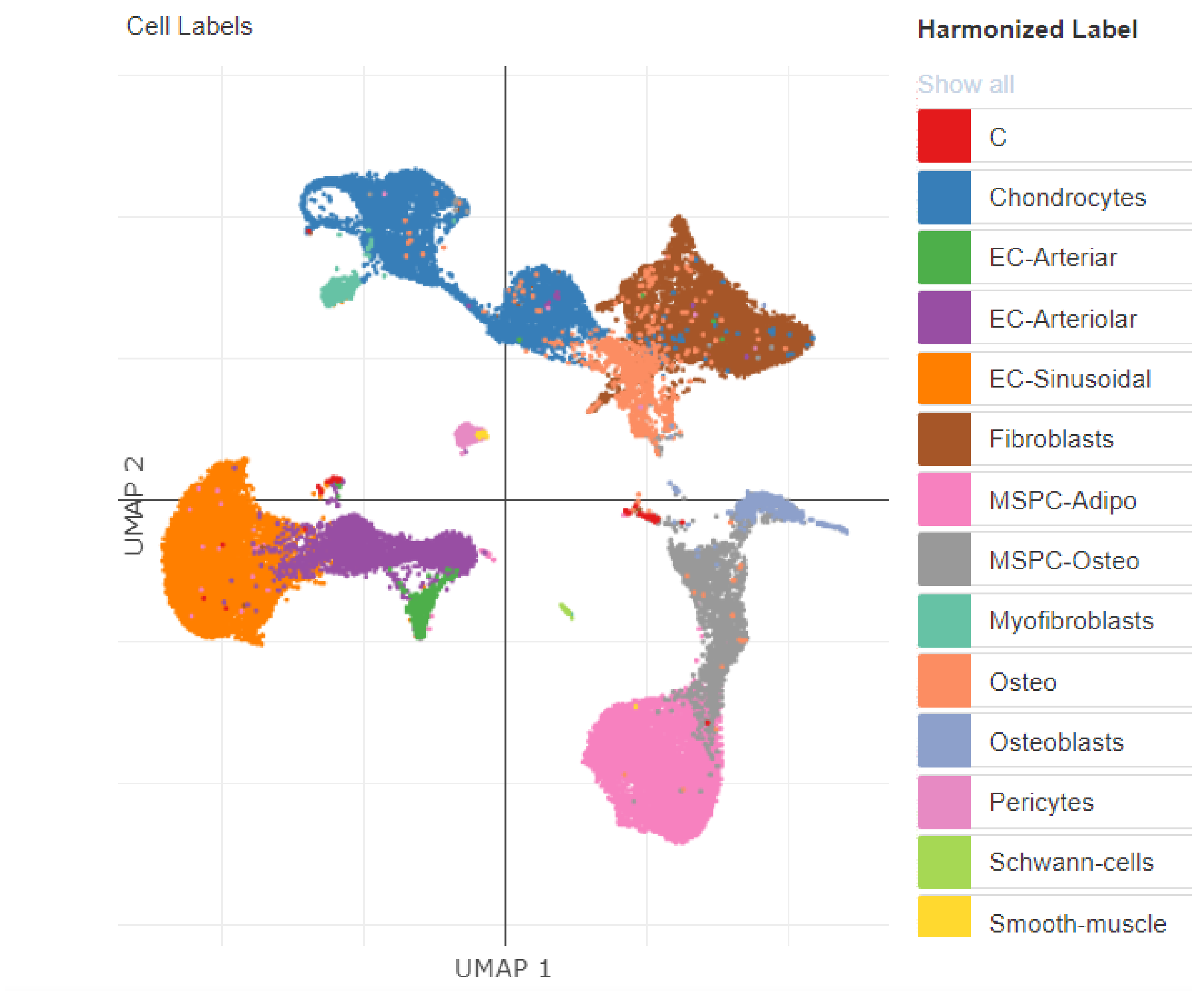

b

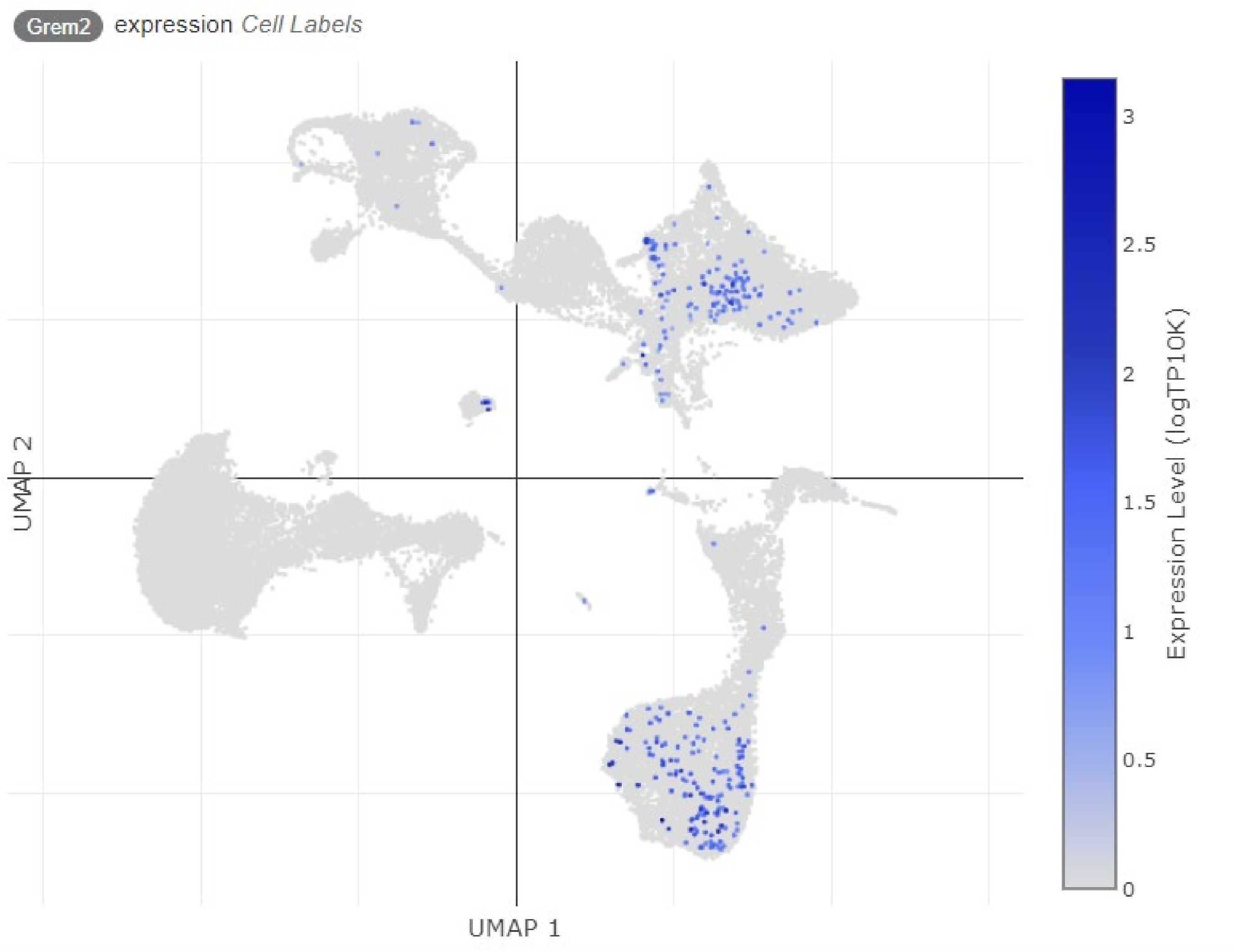

Supplementary Figure 1. *Grem2* expression in cells of the bone marrow niche
Cluster analysis (a), and feature plots of *Grem2* expression (b) in the integrated analysis of published bone marrow niche datasets publicly available at Single Cell Portal (https://singlecell.broadinstitute.org/single_cell/study/SCP1248) (1).

Reference 1: Dolgalev I and Tikhonova AN (2021) Connecting the Dots: Resolving the Bone Marrow Niche Heterogeneity. *Front. Cell Dev. Biol.* 9:622519. doi: 10.3389/fcell.2021.622519

Supplementary Figure 2

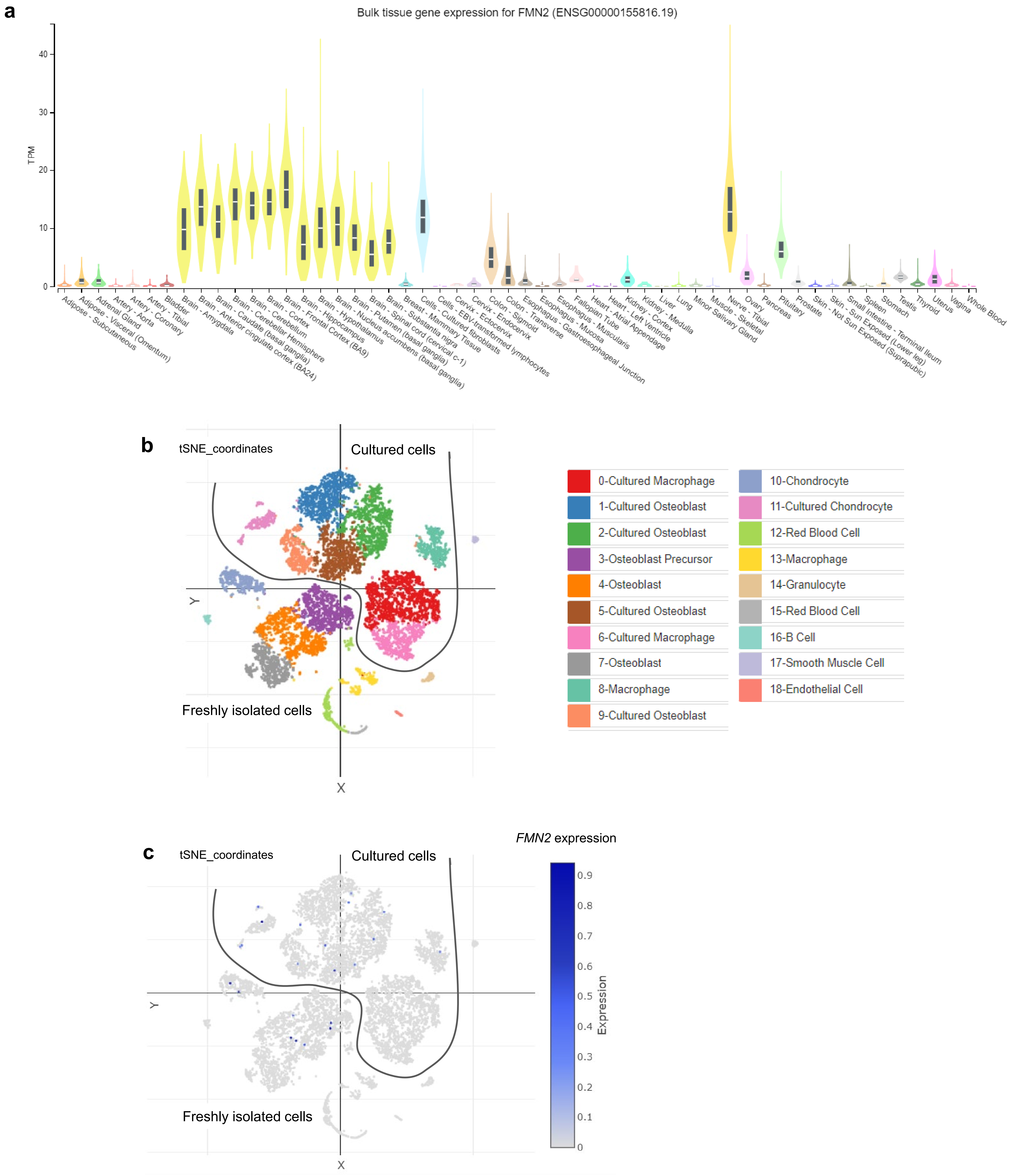

Supplementary Figure 2. *FMN2* expression in human tissues and mouse calvarial cells

a. *FMN2* mRNA expression in multiple (n=54) human tissues as available at the GTEx portal (https://gtexportal.org/home/).

b-c. Cluster analysis (b), and feature plots of *FMN2* expression (c) in freshly isolated primary mouse calvarial cells and cultured calvarial cells published by Ayturk et al (1) and publicly available at Single Cell Portal (https://singlecell.broadinstitute.org/single_cell/study/SCP1337). Freshly isolated cells and cultured cells form separate clusters as indicated in the figure.

Reference 1: Ayturk UM et al (2020) Single-Cell RNA Sequencing of Calvarial and Long-Bone Endocortical Cells. *J Bone Miner Res* 35(10): 1981–1991. doi:10.1002/jbmr.4052.

Supplementary Figure 3

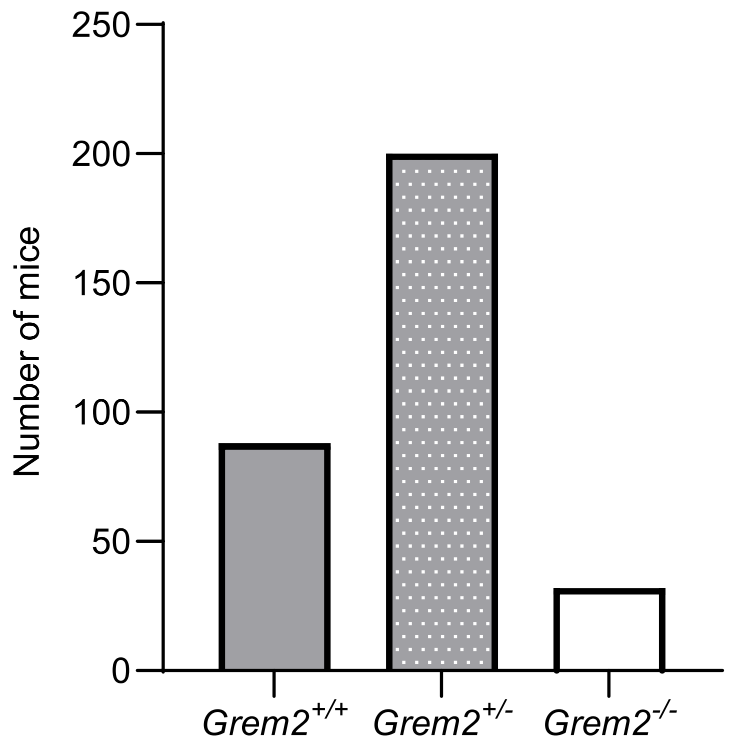

Supplementary Figure 3. Mendelian ratio and survival data
Number of surviving pups from breeding *Grem2*$^{+/-}$ females with *Grem2*$^{+/-}$ males.
Out of 320 pups, 88 were *Grem2*$^{+/+}$, 200 were *Grem2*$^{+/-}$, and 32 were *Grem2*$^{-/-}$.

Supplementary Figure 4

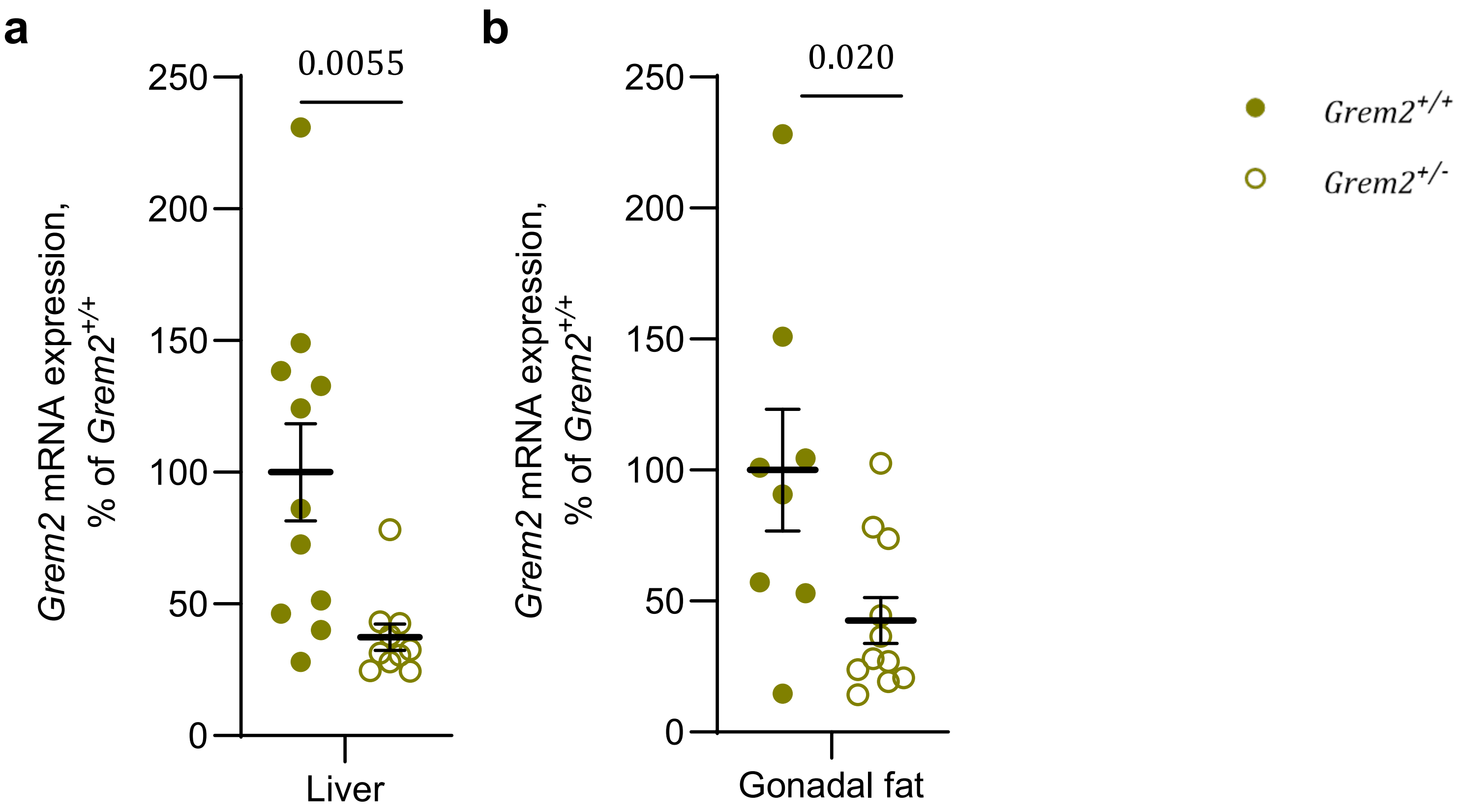

Supplementary Figure 4. *Grem2* mRNA expression in liver (a) and gonadal fat (b)
Grem2 mRNA expression in liver (a) and gonadal fat (b) in 12-weeks-old male *Grem2*$^{+/+}$ (n = 8-11) and *Grem2*$^{+/-}$ (n = 10-11) mice. Individual values are presented with the mean presented as horizontal lines ± standard error of the mean presented as vertical lines. Statistical analyses were performed using two-sided Student's *t* test. A difference was considered statistically significant when $P < 0.05$.

Supplementary Figure 5

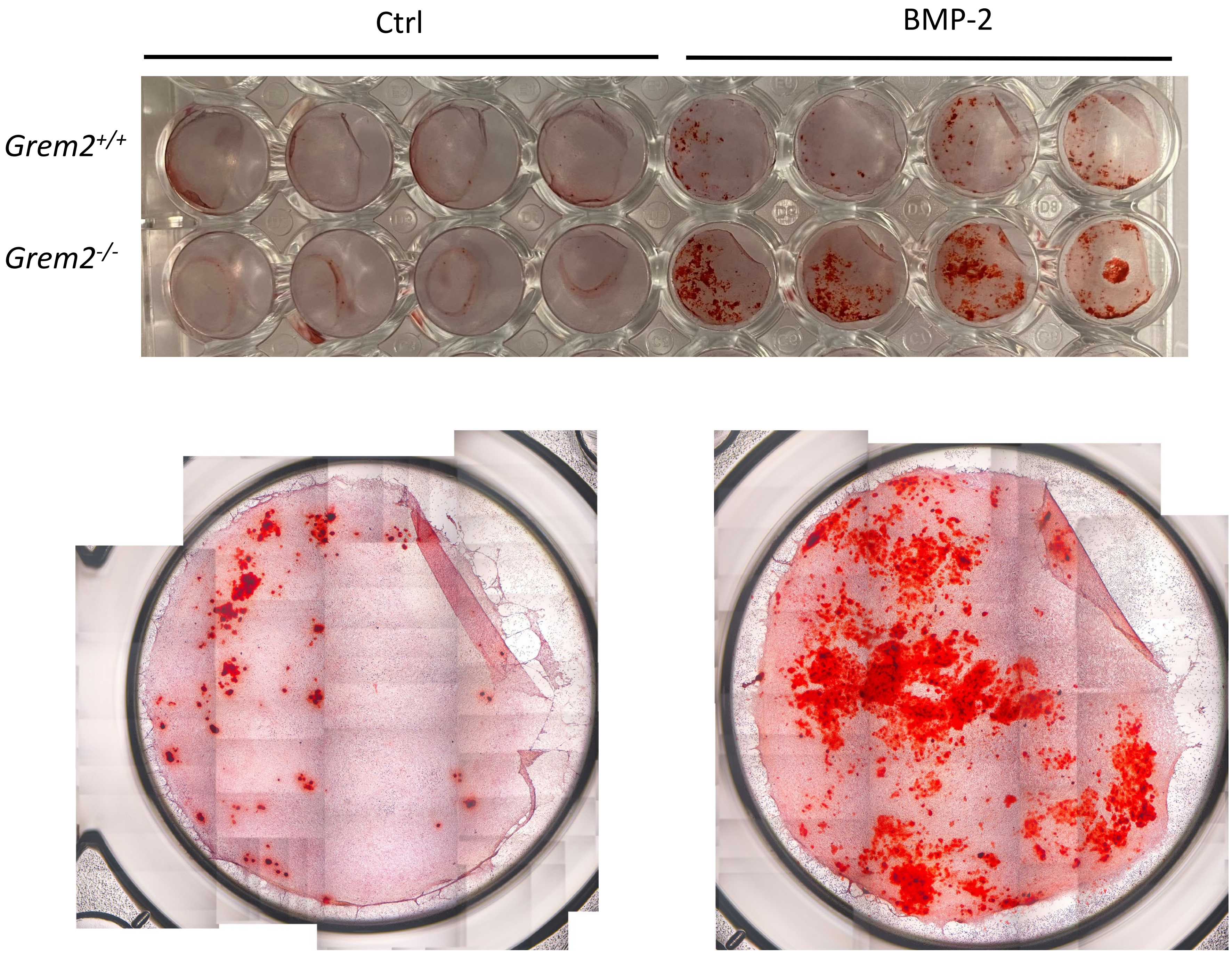

Supplementary Figure 5. Mineralized surface in osteoblasts derived from long bones
Top; Photo of culture plate with alizarin red staining of mineralized surface in cells from *Grem2*$^{+/+}$ mice, compared to cells from *Grem2*$^{-/-}$ mice, stimulated with or without BMP-2. Bottom; Left, cells from *Grem2*$^{+/+}$ mice stimulated with BMP-2; right, cells from *Grem2*$^{-/-}$ mice stimulated with BMP-2. Examples of stiched images of whole wells.
